# How Often Should We Monitor for Reliable Detection of Atrial Fibrillation Recurrence? Efficiency Considerations and Implications for Study Design

**DOI:** 10.1371/journal.pone.0089022

**Published:** 2014-02-12

**Authors:** Efstratios I. Charitos, Paul D. Ziegler, Ulrich Stierle, Derek R. Robinson, Bernhard Graf, Hans-Hinrich Sievers, Thorsten Hanke

**Affiliations:** 1 Department of Cardiac and Thoracic Vascular Surgery, University of Luebeck, Luebeck, Germany; 2 Medtronic Inc., Minneapolis, Minnesota, United States of America; 3 Department of Mathematics, School of Mathematical and Physical Sciences, University of Sussex, Brighton, United Kingdom; University of Minnesota, United States of America

## Abstract

**Objective:**

Although atrial fibrillation (AF) recurrence is unpredictable in terms of onset and duration, current intermittent rhythm monitoring (IRM) diagnostic modalities are short-termed and discontinuous. The aim of the present study was to investigate the necessary IRM frequency required to reliably detect recurrence of various AF recurrence patterns.

**Methods:**

The rhythm histories of 647 patients (mean AF burden: 12±22% of monitored time; 687 patient-years) with implantable continuous monitoring devices were reconstructed and analyzed. With the use of computationally intensive simulation, we evaluated the necessary IRM frequency to reliably detect AF recurrence of various AF phenotypes using IRM of various durations.

**Results:**

The IRM frequency required for reliable AF detection depends on the amount and temporal aggregation of the AF recurrence (p<0.0001) as well as the duration of the IRM (p<0.001). Reliable detection (>95% sensitivity) of AF recurrence required higher IRM frequencies (>12 24-hour; >6 7-day; >4 14-day; >3 30-day IRM per year; p<0.0001) than currently recommended. Lower IRM frequencies will under-detect AF recurrence and introduce significant bias in the evaluation of therapeutic interventions. More frequent but of shorter duration, IRMs (24-hour) are significantly more time effective (sensitivity per monitored time) than a fewer number of longer IRM durations (p<0.0001).

**Conclusions:**

Reliable AF recurrence detection requires higher IRM frequencies than currently recommended. Current IRM frequency recommendations will fail to diagnose a significant proportion of patients. Shorter duration but more frequent IRM strategies are significantly more efficient than longer IRM durations.

**Clinical Trial Registration URL:**

Unique identifier: NCT00806689.

## Introduction

The diagnosis of atrial fibrillation (AF) and the detection of its recurrence after therapeutic interventions are typically performed by electrocardiographic documentation. Reliable and accurate detection of AF recurrence is challenging since its rhythm documentation is discontinuous, whereas the recurrence of AF is often unpredictable [1] and may or may not be accompanied by symptoms [2], [3]. Accurate AF recurrence detection is of importance not only for patient management, but also for the scientific, evidence-based evaluation of therapeutic interventions targeting AF recurrence. Under-detection of AF recurrence introduces a significant external bias, distorts the success rates, and thus affects the scientific evaluation of therapeutic interventions.

Implantable, subcutaneous, leadless as well as intra-cardiac continuous monitoring (CM) devices can reliably detect AF [4]–[8]. However, due to cost considerations and invasiveness of CM, intermittent rhythm monitoring strategies (IRM) of various durations and frequencies are still the most widely used diagnostic modalities for patient monitoring and reporting outcomes. Several groups have compared the efficiency of various IRM strategies with CM devices [5], [6], [9]–[11]. It is now a general consensus that IRM will fail to detect AF recurrence, albeit in a percentage of patients that is unknown and difficult to prospectively estimate [5], [6], [12], [13]. The sensitivity of IRM has been reported to range between 30% and 70% for patients with paroxysmal AF [5], [6], [12], [13]. Our group has previously shown that the success rate of any IRM strategy depends on four factors: the quantitative and temporal characteristics of atrial fibrillation recurrence as well as the frequency and duration of the IRMs performed to detect AF recurrence [5].

The aim of the present manuscript is to investigate the IRM frequencies required to reliably (at least 80% or 95% probability) detect AF recurrence of various AF phenotypes and with various IRM durations. We discuss efficiency considerations as well as implications for clinical trial design.

## Methods

**Figure 1:**
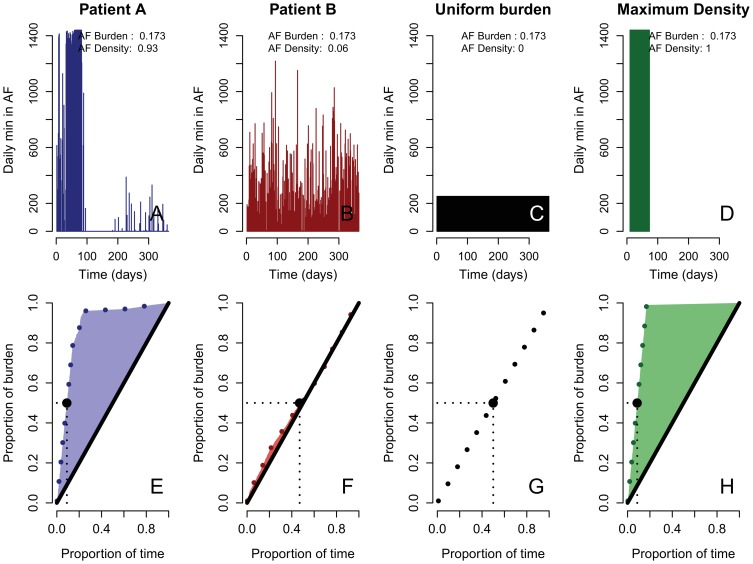
Four examples of different temporal aggregation for the same AF burden (0.173). After reconstruction of the rhythm history (upper panels), the minimum time required for the development of each proportion of the patient's total observed AF burden throughout the monitored period is evaluated (lower panels, dotted lines). AF density is defined as the ratio of the cumulative deviation of the patient's actual burden development (blue or red area) from the uniform burden development (black diagonal line, lower panels and Uniform Burden), to that of the maximum possible burden aggregation for that level of burden (the complete burden as one continuous episode, green area). The black diagonal (lower panels) represents a hypothetical uniform burden aggregation (Uniform Burden). Adapted from [14].

Data acquired from 647 patients monitored with a CM device (Reveal XT cardiac monitor, n = 73; AT500 pacemaker, n = 574; Medtronic, Inc., Minneapolis, MN, USA) were analyzed. Demographics and detailed patient characteristics are displayed in **Table 1**. All patients provided written informed consent for data collection and use. The study has been approved by the local (University of Lübeck) ethics committee (**Clinical Trial Registration URL**: http://www.clinicaltrials.gov **unique identifier**: NCT00806689). The complete rhythm history of each patient was reconstructed from the CM data. AF burden was defined as the proportion of the total monitored time that a patient was in AF. For example, a patient that spends 50% of the time in AF, has an AF burden of 0.5. However, the AF burden alone cannot describe the temporal characteristics of AF recurrence. For example in **Figure 1A and 1B**, the patients A and B have the same overall amount of AF (AF burden  = 0.173) but this AF burden is distributed differently throughout the observation period. To describe the distribution pattern of the AF recurrence we previously proposed the AF density [5], [14], as a quantitative measure of the temporal aggregation of the AF burden consisting of values between 0 (AF burden evenly spread over the observation time) and 1 (maximum possible AF burden aggregation, i.e. the complete AF burden occurs as one continuous episode of AF). Details on the calculation of the AF density have been presented in detail previously [5], [14]. In brief, for each patient, the time course of the AF burden development was analyzed throughout the monitored period (**Figure 1A and 1B** **, for patients A and B respectively**) and the *minimum contiguous monitored time* required for the development of each proportion of the patient's total observed AF burden throughout the monitored period was calculated and evaluated for the overall observation period (blue or red dotted line, **Figure 1E and 1F**, respectively). Patient A (**Figure 1A**) develops 10%, 30%, 50%, 70% and 90% of his total observed burden in 2%, 5%, 9%, 13% and 21% of the total monitored time, respectively (blue dotted line, **Figure 1E**), and most of the AF recurrence and burden development occurs between days 30 and 80. This information on the temporal burden development of patient A is displayed in **Figure 1E**. In contrast, patient B (**Figure 1B**) develops 10%, 30%, 50%, 70% and 90% of his total observed burden in 6%, 24%, 48%, 71% and 89% of the total monitored time respectively, since the total burden is spread over more days and as such each day contributes less to the total burden development. This information on the temporal burden development of patient B is displayed in **Figure 1F**. The black diagonal line (**Figure 1E and 1F**) represent the hypothetical development of the patient's AF burden if this burden had been uniformly distributed over the monitored time (same AF duration every day throughout the observation period, uniform burden, **Figure 1C and 1G**). The green dotted line (**Figure 1H**) represents the burden development of a hypothetical patient in which the AF burden (equal in amount to that of patient A and B) occurs as one single continuous episode (maximum density, **Figure 1D**).

**Table 1:** 

	Total	%
**Male**	376	58.1
**Age**	68.9±12.3	
**Follow up (mean ± sd, range; years)**	1.1±0.4, 0.1–3.7	
**History of Atrial Arrhythmia**		
Atrial Tachycardia	114	17.6
Atrial Flutter	176	27.2
Paroxysmal AF	475	73.4
Persistent AF	32	4.9
Long lasting persistent AF	35	5.4
**History of Cardioversion**	18	2.8
**Cardiovascular History**		
Ischemic Heart Disease	99	15.3
Coronary Artery Disease	220	34.0
Cardiomyopathy	64	9.9
Hypertension	405	62.6
**History of ablation for AF**		
Cox-Maze III	17	2.6
Left sided only	53	8.2
AV Node Ablation	28	4.3
Other	71	11.0
**History of Cardiac Surgery**		
CABG	113	17.5
MVR	45	7.0
AVR	41	6.3
TVR	7	1.1
Asc. Aorta Replacement	9	1.4
PVR	1	0.2
**NYHA Class**		
I	331	51.2
II	234	36.2
III	67	10.4
IV	3	0.5
**Pacing Indication**		
AV-Block	85	13.1
Sinus node dysfunction	397	61.4
Other	41	6.3
**Arrhythmia related medication**		
Class I	89	13.8
Class III	251	38.8
Beta-Blocker	212	32.8
Calcium Channel Blocker	56	8.7
Digoxin	144	22.3

Patient demographics: AF: atrial fibrillation; AV: atrioventricular; CABG: coronary artery bypass grafting; MVR: mitral valve replacement/repair; AVR: aortic valve replacement/repair; TVR: tricuspid valve replacement/repair; PVR: pulmonary valve replacement; NYHA: New York Heart Association.

For the calculation of the AF density as a measure of temporal AF burden aggregation, the patient's complete rhythm history is scanned and the *minimum contiguous time* required to develop each proportion *p* of the patients total burden *b* is calculated (red and blue dotted line, **Figure 1E and 1F**). We define as *AF density*, the ratio of the cumulative deviation of the patient's actual burden development (blue or red area, **Figure 1E and 1F**, respectively) from the hypothetical uniform burden development (black diagonal line, **Figure 1E and 1F**), to that of the hypothetical maximum possible burden aggregation for that level of burden from the hypothetical uniform burden development (the complete burden as one continuous episode, green area, **Figure 1H**). The black diagonal (**Figures 1E, 1F, 1G, 1H**) represents a hypothetical uniform burden aggregation (**Figure 1C**).

For the numerical evaluation of the AF density we define: For a patient with a total AF burden *b* (expressed as the proportion of the observation time the patient is in AF), who is monitored for time *T*, we denote the *minimum* contiguous monitored time throughout the monitored period *T* required for the development of a proportion *p* of the patient's total observed burden (*b*) as 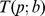. This time, expressed as the proportion of the total observed time *T*, is
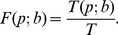
Figures such as **1E** and **1F** are plots of *p* against *F(p;b)* for 0≤p≤1.

The cumulative deviation of the patient's actual burden development from the hypothetical uniform burden development (black diagonal line, **Figure 1E, 1F, 1G, 1H**) can be evaluated as 
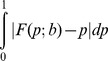
and is equal to the shaded area **(blue shaded area for patient A,** **Figure 1E** **; red shaded area for patient B;** **Figure 1F** **)**. For the hypothetical patient with maximum temporal aggregation of burden *b* (the complete burden as one continuous AF episode) the cumulative deviation of this patient's burden development (green line, **Figure 1H**) from the hypothetical uniform burden development (black diagonal line, **Figure 1G**) is evaluated as
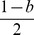
and is equal to the green shaded area (**Figure 1H**). AF density for patients A and B is then the ratio of the blue or red areas respectively, to the green shaded area and is defined as:
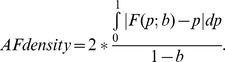
The AF density as the ratio of the above mentioned areas is therefore a dimensionless quantity and assumes values between 0 and 1, with values close to 0 denoting low burden aggregation (AF burden evenly spread throughout the monitored period, **Figure 1B & 1F** as well as **Figure 1C & 1G**), whereas values close to 1 denote maximal burden temporal aggregation (the complete AF burden occurring as a single continuous episode or “a block of AF”, **Figure 1A & 1E** as well as **Figure 1D & 1H**).

After reconstruction of each patient's complete rhythm history, we used computationally intensive simulation to repeatedly simulate IRM of various durations (1, 7, 14, 30 days) and frequencies (1, 2, 3, …,12 as appropriate) in every patient. The stochastic process of the simulation procedure was the following: After reconstructing the rhythm history of every patient *j*, monitored for a total of *g* days, we defined the sample space Ω_kj_ = {1, 2, …, g−k+1} to be the set of possible days that a *k*-day intermittent monitoring session could be started (for *k* = 1, 2, 3, …, 30). A *k*-day monitoring session starting on day i ∈ Ωkj therefore included the following associated monitored days: {i, i+1, …, i+k−1}. To simulate a strategy of n independent *k*-day monitorings of patient *j*, n elements were selected at random from Ω_kj_, except that elements were rejected if their monitored days intersected with the monitored days of previously selected elements. This was performed for all patients of the study population, for monitoring durations of *k* = {1,2,3…,30} days, and for strategies of n = {1, 2, 3, …, 12} monitorings per year, where appropriate. In every simulated IRM, AF was deemed to have been successfully identified if it was observed on at least one of the monitored days. The simulations were performed in all patients, for every IRM strategy (all IRM frequencies and IRM durations) sufficient times (>10^5^) to allow stabilization of the inferred probability of AF burden recurrence.

**Figure 2:**
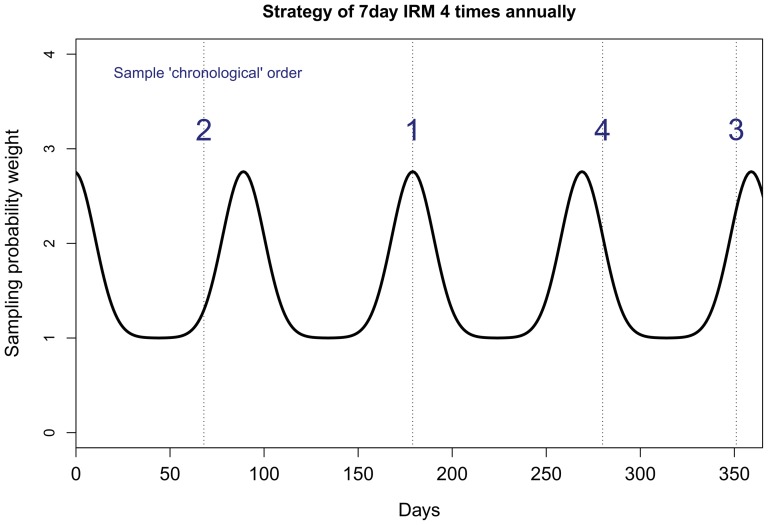
Illustration of the simulation procedure: Initially, the “first day” of the first monitoring period (IRM) was chosen at random, and then the subsequent k-1 days were counted to make a k day monitoring period (IRM). In this example the 7-day IRM would be on days 179–185. Any future sampling that includes days 173–191 will be rejected (any 7 day IRM starting on these days would intersect with the sampled IRM on days 179–185). Second, the simulation procedure scans the total observation period of the patient and at even intervals (based on the pre-specified IRM frequency) attaches weights at the following days: first sampled day±k*(365/sampling strategy frequency), where k = {1,2,3,4, …, sampling strategy frequency}. In this example, the algorithm attaches higher sampling weights at days 9, 99, 279, 370. Gaussian smoothing was used to construct a smoothed sampling weight curve such that the zeniths have about 3 times higher probability to be sampled than the nadirs. Attaching higher sampling probability weights at even intervals mimics the follow-up strategy of clinical trials, while also simultaneously allowing some randomness (just as in real life follow-up examinations, patients often are seen slightly before or after their nominal follow-update). Thereafter the simulation proceeds with sampling from the weighted sample space.

In order to mimic the typical timely follow-up strategy employed in clinical trials, the simulation worked as follows: First, the “first day” of the first monitoring period (IRM) was chosen at random, and then the subsequent *k-1* days were counted to make a *k* day monitoring period (IRM). An example is illustrated in **Figure 2** for the simulation of 7-day monitoring. The first sampled day happened to be on day 179, therefore the 7-day IRM would be on days 179–185. Any future sampling that includes days 173–191 will be rejected (i.e., any 7-day IRM starting on these days would intersect with the sampled IRM on days 179–185).

Second, the simulation procedure then scans the total observation period of the patient and at even intervals (based on the pre-specified IRM frequency) attaches weights at the following days: first sampled day±k*(365/sampling strategy frequency), where k = {1,2,3,4, …, sampling strategy frequency}. In the example shown in **Figure 2**, the algorithm attaches higher sampling weights at days 9, 99, 279 and 370. Gaussian smoothing was used to construct a smoothed sampling weight curve (**Figure 2**) such that the zeniths have about 3 times higher probability to be sampled than the nadirs. Attaching higher sampling probability weights at even intervals mimics the follow-up strategy of clinical trials, while also simultaneously allowing some randomness (just as in real life follow-up examinations, patients often are seen slightly before or after their nominal follow-update). Thereafter the simulation proceeds with sampling from the weighted sample space. For every patient and for every IRM duration and frequency, sufficient simulations were carried out (>10^5^) to estimate the probability of AF detection accurately. The required IRM frequency to achieve at least 80% (*f_80%_*) and at least 95% (*f_95%_*) probability of AF detection, could then be determined for each patient and IRM duration. All simulated IRM was of the continuous recording type and patient compliance was assumed to be 100%.

Response surface models were used to evaluate the dependency of the probability of AF detection (*f_95%_, f_80%_*) on AF burden and density. These models are of the form:
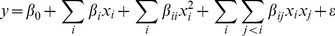



In modeling *f_95%_* and *f_80%_* it was found appropriate to use its natural logarithm, so that *y*  =  ln(*f_95%_* or *f_80%_*), *x*_1_ = AF burden, *x*_2_ = AF density. Separate models were used for the most commonly used IRM durations (24 hours, 7 days, 14 days and 30 days). Backwards stepwise elimination was used to derive the final models which are presented in **Table 2**.

**Table 2:** Regression (response surface models) of the AF characteristics (AF burden, AF density) on the IRM frequencies required to achieve 95% probability of AF recurrence detection.

Factor	Coefficient (mean±SE)	p
**24h IRM model (R^2^ = 70.4%)**		
Intercept	−2.01±0.08	<0.001
AF Burden	−4.82±0.40	<0.001
AF Density	1.3±0.11	<0.001
AF Burden^2^	2.13±0.42	<0.001
**7d IRM model (R^2^ = 70.2%)**		
Intercept	0.88±0.07	<0.001
AF Burden	−5.09±0.35	<0.001
AF Density	1.90±0.10	<0.001
AF Burden^2^	3.03±0.39	<0.001
**14d IRM model (R^2^ = 69.4%)**		
Intercept	0.52±0.06	<0.001
AF Burden	−4.40±0.32	<0.001
AF Density	1.89±0.10	<0.001
AF Burden^2^	2.67±0.37	<0.001
**30d IRM model (R^2^ = 60.4%)**		
Intercept	0.22±0.10	0.03
AF Burden	−2.52±0.27	<0.001
AF Density	1.11±0.39	<0.001
AF Burden^2^	1.32±0.31	<0.001

To restore normality, the natural logarithm of the required IRM frequency was regressed. Separate models were fit for the four IRM modalities (24-hour, 7-day, 14-day, and 30-day). *SE: standard error, IRM: intermittent rhythm monitor, AF: atrial fibrillation*.

All simulations were performed on multiple parallel AWS (Amazon Web Services, WA, USA) Elastic Cloud Computing high performance compute clusters (cc8.xlarge) running Ubuntu Server Linux 12.04.01 LTS (AWS Cluster Instances). A primer on the infrastructure and use of high performance cloud computing for biomedical research and simulation studies has been published elsewhere [15], [16]. The statistical analyses and simulations were performed with R version 2.15.3 [17]. The P values of 2-sided tests are reported.

## Results

### Estimation of the probability of AF detection with an intermittent monitoring strategy

**Figure 3:**
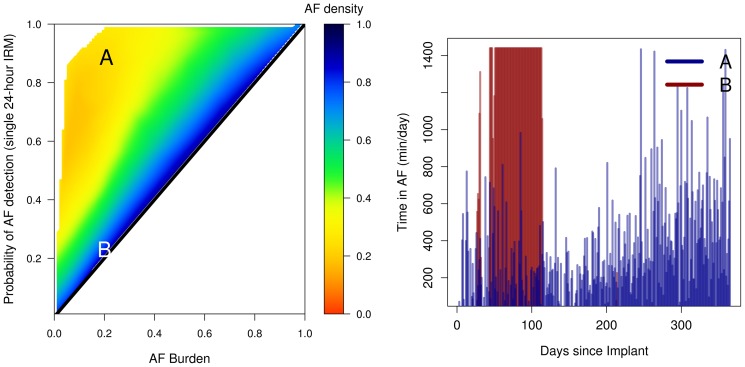
The effect of AF burden and AF density on the probability of AF detection with a single 24-hour IRM (left panel) for the AF recurrence pattern of two example patients (right panel). For any given AF burden b, observed during any time frame, the probability of successful identification using a given IRM duration ranges between ≅b and 1. The range [≅b,1] depends on the temporal aggregation of the AF recurrences (AF density). If the AF occurs as one episode, the probability of AF detection is ≅b (**Patient B**), whereas if the AF recurrence is uniformly spread throughout the observation time the probability of AF recurrence detection is 1 (**Patient A**). *IRM: intermittent rhythm monitor, AF: atrial fibrillation*.

For a single IRM of *n*-days duration and for any given AF burden *b*, observed during any sufficiently large time frame *t (t>>n)*, the probability of successful AF detection ranges between ≅*b* and 1. Its exact value within the range [≅*b*,1] depends on the temporal aggregation of the AF recurrence [5]. If the AF occurs as one episode, the probability of AF detection is ≅*b* (**Figure 3** **, Patient B**), whereas if the AF recurrence is uniformly distributed throughout the observation time the probability of AF recurrence detection is 1 (**Figure 3** **, Patient A**). We have shown previously that the AF density can efficiently describe the temporal aggregation and thus the recurrence pattern of the AF burden [5], [14]. For the IRM durations examined in the present work, AF burden and AF density had a significant effect in determining the probability of successful AF detection with a single random IRM (for all IRM durations: p<0.0001 for AF burden and AF density, R^2^ >85%). Using computationally intensive simulation, similar graphs and relationships can be obtained with IRMs of any duration and/or frequency.

**Figure 3 left panel** shows the effect of AF burden and density on the probability of AF detection of a single 24 hour IRM. Both Patient A and B have been continuously monitored for 364 days and both have an AF burden of 0.2, however the temporal AF burden distribution differs (**Figure 3 right panel**). Patient A has a low temporal AF aggregation (AF spread throughout the observation period with an AF density of 0.28), whereas patient B has the majority of the AF burden developed only between day 50 and 114 (AF density = 0.98). Due to the high temporal aggregation (increased AF density), the probability that a random 24-hour IRM will detect AF recurrence in patient B is 0.23. In contrast, in patient A, AF recurrences are distributed over almost every day of the entire monitored time and thus the probability of AF recurrence detection is much higher, 0.9.

### Frequency of IRM required to reliably detect AF recurrence according to AF burden and AF density

**Figure 4:**
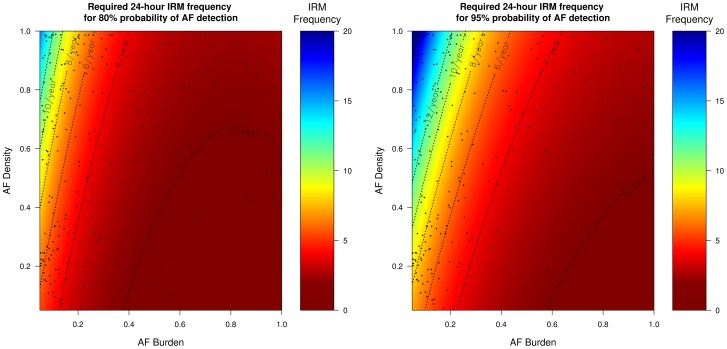
Required 24-hour IRM frequency to achieve 80% (left) and 95% (right) probability of AF detection. The black dots represent our patient population. *IRM: intermittent rhythm monitor, AF: atrial fibrillation*.

Since the probability of successful AF identification depends on the AF characteristics (AF burden and AF density), inferences can be drawn regarding how many times IRM should be performed in order to reliably (probability >95%) detect AF recurrence. **Table 2** presents the results of the regression analyses, of the AF characteristics on the required IRM frequency to achieve >95% probability for AF detection. These results are more clearly visualized in **Figures 4** **–** **7** which present the frequency of IRM required to detect AF recurrence with 80% and 95% probability as a function of the AF characteristics (AF burden and AF density). For all IRM durations, the IRM frequency required to detect AF recurrence with a 95% probability increases as the AF burden decreases and as the AF density increases (AF burden less spread throughout the observation time) (**Table 2**).

**Figure 5:**
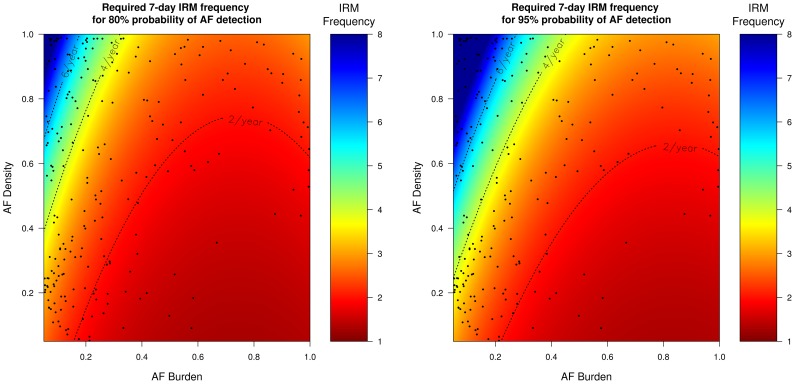
Required 7-day IRM frequency to achieve 80% (left) and 95% (right) probability of AF detection. The black dots represent our patient population. *IRM: intermittent rhythm monitor, AF: atrial fibrillation*.

**Figure 6:**
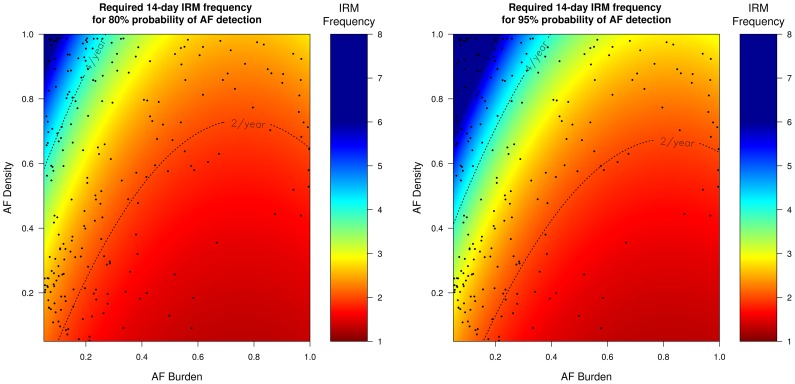
Required 14-day IRM frequency to achieve 80% (left) and 95% (right) probability of AF detection. The black dots represent our patient population. *IRM: intermittent rhythm monitor, AF: atrial fibrillation*.

**Figure 7:**
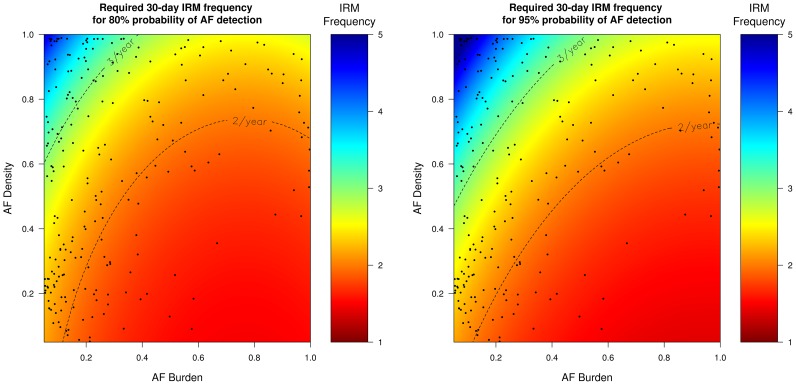
Required 30-day IRM frequency to achieve 80% (left) and 95% (right) probability of AF detection. The black dots represent our patient population. *IRM: intermittent rhythm monitor, AF: atrial fibrillation*.

**Figures 4** **–** **7** indicate that strategies of twelve 24-hour, six 7-day, four 14-day and three 30-day per year will be able to detect on average 96%, 95%, 91%, 90% of all AF recurrence phenotypes (AF burden/AF density combinations) with a probability of 95% (area below the respective IRM frequency dotted lines, **Figure 4** **–** **7**). In our patient population, which had a mean burden of 0.12±0.22, the above mentioned IRM strategies would succeed in diagnosing AF recurrence in 83%, 77%, 70%, 68% of these patients (dots below the respective IRM frequency lines, **Figure 4** **–** **7**). Patients with even lower burdens and/or higher densities (dots above the respective IRM frequency lines, **Figure 4** **–** **7**) will require more aggressive monitoring strategies which increases the likelihood of poor compliance.

### Time efficiency as a function of AF burden and AF density

**Figure 8:**
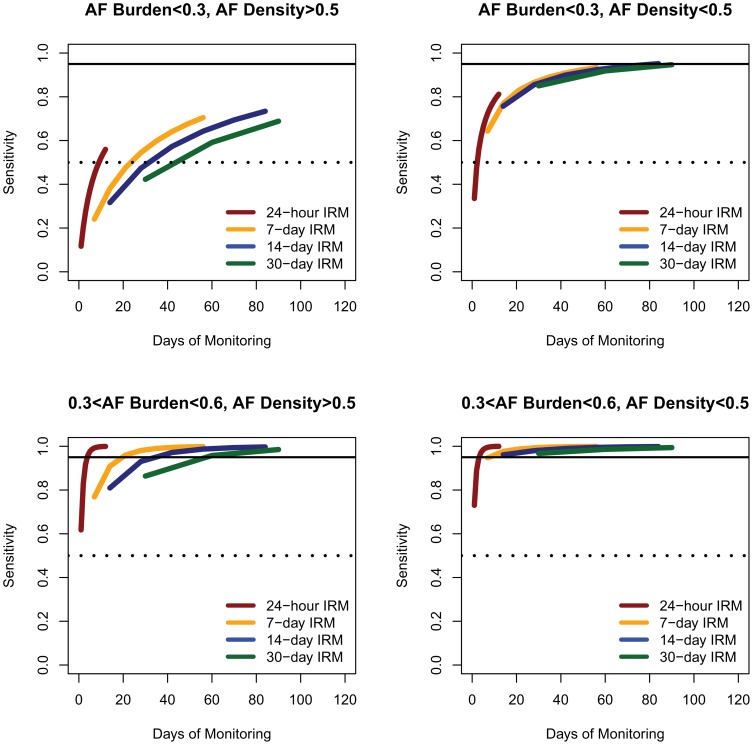
Time efficiency (sensitivity obtained per monitored day) of IRM strategies. For the same amount of total monitored time, shorter IRM durations result in higher sensitivities. The dotted and solid horizontal line represents sensitivities of 0.5 and 0.95 respectively. *IRM: intermittent rhythm monitor, AF: atrial fibrillation.*

The time efficiency (sensitivity obtained per day of IRM) was dependent on both AF characteristics (AF burden and AF density) and IRM characteristics (IRM duration and IRM frequency; p<0.001 for all). For the same overall IRM duration, the 24-hour IRM results in higher sensitivity per unit of monitored time than prolonged IRM durations (**Figure 8**). As **Figures 4** **–** **7** show, strategies of twelve 24-hour, six 7-day, four 14-day and three 30-day per year will be able to detect on average 96%, 95%, 91% and 90% of all AF recurrence phenotypes (AF burden/AF density combinations) with a probability of 95%, or 83%, 77%, 70% and 68% of our patient population, respectively. However to obtain these sensitivities, these strategies would require a total monitoring time of 12, 42, 56, and 90 days for the 24-hour, 7-day, 14-day and 30-day IRM, respectively.

## Discussion

Currently the most widely used diagnostic modality for the detection of AF recurrence is IRM. However, in contrast to other diagnostic examinations in medicine which are required to have the highest possible sensitivity and specificity, the absence of non-invasive and cost efficient alternatives has made IRM a standard for AF recurrence detection and rhythm follow-up even though its sensitivity as a diagnostic test is strikingly low [5], [6], [11]–[13], [18]. Since the identification of AF recurrence is seminal importance for accurate patient management and for the scientific evaluation of therapeutic AF interventions, we sought to determine the frequency of IRMs required to identify AF recurrence with 80% and 95% probability.

### Determining the probability of AF detection

Our results indicate that the quantitative AF characteristics (AF burden and AF density) as well as the IRM strategy characteristics (IRM frequency and IRM duration) can determine the probability of successful AF identification using IRM. **Figure 3 Left Panel** shows the interrelation between AF burden and AF density in determining the probability of AF identification with a single random 24-hour IRM. Similar relationships can be obtained for other IRM durations as well as IRM frequencies. Our analyses show that the probability of AF identification increases as the AF density decreases (AF recurrence spread more evenly throughout the observation time) and/or AF burden increases. The influence of AF density on the AF detection probability for any IRM is of critical importance at lower burdens (<0.5) and diminishes progressively at higher (>0.5) AF burdens (**Figure 3 Left Panel**).

Current guidelines and consensus statements address the need for intermittent monitoring for the detection of AF recurrence [12], [13], [18]. Although most recommendations suggest an IRM monitoring strategy between two and four 24-hour IRM per year for the detection of AF recurrence, most statements simultaneously note that these strategies have a limited sensitivity and a significant proportion of patients with AF recurrence will not be detected. A speculative estimate is that IRM strategies may detect up to 70% of patients with AF recurrence and may have a negative predictive value of up to 50% [12], [13], [18]. However these estimates, which stem from published studies, cannot be reliably transferred to other studies or other prospectively recruited populations. Our results point out that the sensitivity and thus the required frequency to reliably detect AF recurrence, depends largely on the AF and IRM characteristics and may deviate from the above estimates. For example, as **Figure 4** illustrates, a strategy of four 24-hour IRMs would fail to identify 75% of our patient population with otherwise proven AF recurrence (**Figure 4**, percentage of black dots being above and to the left of the 4/year dotted line).

### Do longer IRM durations lead to increased AF detection?

In light of the limitation of short duration IRM, longer IRM durations have been proposed for more accurate AF recurrence detection. We and others have previously seen that on average, longer duration IRM indeed results in more accurate AF recurrence detection [5], [6]. However, longer IRM pose two distinct disadvantages:

First, the benefit of longer IRM is not linear with respect to the monitoring time. Especially in patients with high density AF, longer IRM may result in little or no practical benefit in terms of the probability of AF recurrence detection [5], [14]. The efficiency of longer IRMs diminishes as the monitoring time increases. This is presented in **Figure 8**. When considering the monitored days (the number of days the patient wears the IRM), the 24-hour IRM achieves higher sensitivities, with less monitoring days than all other prolonged IRM durations. This stems from the fact that an *n*-day IRM will only be able to detect AF recurrence taking place within the *n* consecutive days from the start of the IRM, whereas, 24-hour IRMs are performed *n* times at *n* different time points during the follow-up period and thus will be able on average to detect AF recurrence with greater probability. This is especially important for patients with paroxysmal AF as it has been shown that paroxysmal AF frequently recurs in clusters (higher AF density, low AF burdens) [1] thus monitoring for AF at *n* different time points is more advantageous than monitoring *n* consecutive days.

Second, there is now significant evidence that longer IRM durations adversely affect patient compliance[12]. Reported causes of patient non-compliance with scheduled monitoring include skin irritation, interference with showering or exercise, and feelings of self-consciousness when wearing the monitoring equipment in public[19]. A recent study with an external arrhythmia monitoring patch reported that the device fell off in 22% of patients and resulted in a mean wear time of 7.9±1.8 days instead of the planned 14 days[20]. Kamel at al. reported that patients randomized to monitoring via mobile cardiac outpatient telemetry wore the monitors for 64% of the assigned days and that 25% of patients were not compliant at all with the scheduled monitoring [21]. In another study which utilized the same monitoring technology in 19 patients who recently underwent catheter ablation for AF, only 53% of patients complied with the scheduled monitoring[22]. Shorter, but more frequent IRMs tend to have much higher patient compliance and may result in better AF recurrence detection under real life conditions. Additionally, shorter IRMs performed more frequently may lead to less patient discomfort and disruption of daily activities.

### Intermittent rhythm monitoring in the era of continuous monitoring

Although an aggressive monitoring strategy of twelve 24-hour IRM per year may reliably detect the great majority of patients (83.4% of our patient population, 96.0% of the AF burden/AF density plane, **Figure 3**), it should be noted that there is still an element of chance in this process (80% or 95% probability of AF detection) and some patients may be misclassified. This seems to be of importance when designing clinical studies, especially when the outcome of interest is of low incidence. Furthermore, such aggressive strategies still require considerable amount of resources as well as patient compliance and physician commitment.

Continuous monitoring is an attractive alternative to IRM. Indeed, several studies lately have recently surfaced in which follow-up of patients usually after therapeutic interventions for AF is being performed using implantable subcutaneous monitoring devices or using the readings of intra-cardiac devices capable of detecting and recording AF recurrence. A great advantage of this approach is that these devices not only allow much more accurate AF recurrence detection but also can provide the calculation of quantitative AF indices (e.g. AF burden) and may allow better understanding of the AF recurrence dynamics. However, the implantation of leadless CM requires a minor surgical procedure which may carry a small risk of infection or patient discomfort. CM has a higher initial cost, however rhythm disclosure and reporting can be reported remotely via telemetry and patient visits can be scheduled on an “as needed” basis [23]. Additionally, the current generation of implantable leadless CM devices can provide rhythm disclosure for at least 3 years after implantation, a fact which may increase the cost efficiency of CM over that period. Although intra-cardiac and leadless rhythm monitors may fail to detect or may misclassify a small number of AF episodes, these erroneous episodes tend to be very brief in duration and therefore have minimal impact on the overall AF burden measurement. Several studies have shown that these devices can quantify AF burden with ≥98.5% accuracy [4], [8].

Nevertheless there is currently no evidence that in patients with AF, continuous rhythm monitoring can improve patient specific outcomes compared to IRM. More data are required to evaluate the impact of CM on patient specific outcomes. However, in the setting of clinical trials, the complete and accurate rhythm disclosure that CM devices provide can lead to a more accurate understanding and scientific evaluation of treatments for AF.

## Limitations

Our methodology does not allow and does not take into consideration patient symptoms which may help guide the AF follow-up. However, numerous studies have shown that symptoms have a low sensitivity and specificity [3], which may or may not lead to better and more reliable AF recurrence detection. Moreover, for the scientific evaluation of AF treatments even with the presence of symptoms, and because of their low sensitivity and specificity, AF recurrence still should be electrocardiographically documented. Additionally, recent evidence shows that after invasive AF treatments the ratio of asymptomatic to symptomatic AF episodes increases [2], [12]. Therefore, although a limitation, we believe that not accounting for patient symptoms does not limit the validity and applicability of our findings. Additionally, our analysis assumed 100% patient compliance with all simulated IRM strategies. In reality, reduced patient compliance with more intensive external monitoring would further diminish the ability of IRM to detect AF recurrences.

## Conclusion

Reliable AF recurrence detection requires higher IRM frequencies than currently recommended, especially for patients with low (<0.5) AF burdens. Current IRM frequency recommendations will fail to identify AF recurrences in a significant – albeit difficult to prospectively estimate - proportion of patients. Shorter duration IRM performed more frequently during the follow-up period are significantly more efficient with respect to time and probably patient compliance than longer duration IRMs for the same amount of monitored time. For the scientific evaluation of AF treatments and confident detection of AF recurrence, especially in the setting of clinical trials, continuous monitoring should be considered.
